# RAS pathway regulation in melanoma

**DOI:** 10.1242/dmm.049229

**Published:** 2022-03-02

**Authors:** Amira Al Mahi, Julien Ablain

**Affiliations:** Centre de Recherche en Cancérologie de Lyon, Centre Léon Bérard, INSERM U1052 CNRS UMR5286, Tumor Escape, Resistance and Immunity Department, 69008 Lyon, France

**Keywords:** Cancer genetics, Melanoma, RAS pathway, Signaling, Targeted therapies

## Abstract

Activating mutations in RAS genes are the most common genetic driver of human cancers. Yet, drugging this small GTPase has proven extremely challenging and therapeutic strategies targeting these recurrent alterations have long had limited success. To circumvent this difficulty, research has focused on the molecular dissection of the RAS pathway to gain a more-precise mechanistic understanding of its regulation, with the hope to identify new pharmacological approaches. Here, we review the current knowledge on the (dys)regulation of the RAS pathway, using melanoma as a paradigm. We first present a map of the main proteins involved in the RAS pathway, highlighting recent insights into their molecular roles and diverse mechanisms of regulation. We then overview genetic data pertaining to RAS pathway alterations in melanoma, along with insight into other cancers, that inform the biological function of members of the pathway. Finally, we describe the clinical implications of RAS pathway dysregulation in melanoma, discuss past and current approaches aimed at drugging the RAS pathway, and outline future opportunities for therapeutic development.

## Abstract (precis)

**Summary:** This Review describes the molecular regulation of the RAS pathway, presents the clinical consequences of its pathological activation in human cancer, and highlights recent advances towards its therapeutic inhibition, using melanoma as an example.

## Introduction

**Fig. 1.**
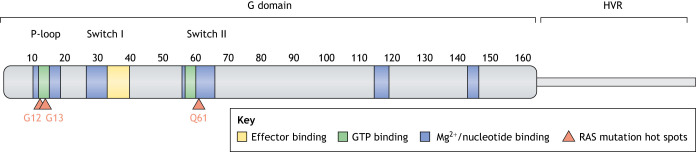
**RAS structure and functional domains.** The 164 N-terminal residues constitute the G domain, which is involved in GTP binding, effector interactions and Mg^2+^/nucleotide binding. Its Switch I and Switch II regions (aa 30-38 and 60-67, respectively) mediate protein interactions, and the P-loop region (aa 1-17) binds phosphate groups. The C-terminal end corresponds to the hypervariable region (HVR) of RAS, containing sequences that interact with the membrane. Indicated are amino acid positions G12, G13 and Q61 that are frequently mutated in cancer (G12D, G12V, G12C, G13D and Q61R), and account for 70% of all RAS mutants in patients (Prior et al., 2020).

RAS proteins are small GTPases that can adopt either of two conformations: an inactive GDP-bound state or an active GTP-bound state (Bourne et al., 1990). The activation status switches via guanine nucleotide exchange, with GTP hydrolysis reverting to the inactive GDP-bound conformation. This switch is tightly controlled by multiple regulators and involves profound conformational changes in the switch I and switch II regions of the highly conserved catalytic G-domain (Milburn et al., 1990) (Fig. 1). The C-terminal domain of RAS proteins comprises a hypervariable region (HVR) that differs between isoforms and is essential for their association with the plasma membrane (Willumsen et al., 1984). Over 150 RAS genes have been identified in mammalian genomes (Goitre et al., 2014; Wennerberg et al., 2005). Among these, the most studied are *KRAS* (Kristen-RAS) (Der et al., 1982), *HRAS* (Harvey-RAS) (Parada et al., 1982; Santos et al., 1982) and *NRAS* (neuroblastoma-RAS) (Hall et al., 1983; Shimizu et al., 1983). *KRAS* and *HRAS* were initially identified in cancer cells as the human orthologs of those proto-oncogenes responsible for the initiation of sarcoma in rats infected with two cancer-causing viruses, while *NRAS* was discovered shortly after by homology with *KRAS* and *HRAS* (Malumbres and Barbacid, 2003). The RAS proteins regulate cellular responses to growth factors by activating multiple molecular cascades that transduce signals from the membrane to the nucleus and, thus, have crucial roles in many biological processes, including cell proliferation, differentiation, migration and apoptosis (Campbell et al., 1998).

More than 30% of all human cancers harbor oncogenic mutations in RAS genes (Hobbs et al., 2016). In melanoma, an aggressive form of skin cancer caused by the malignant transformation of melanocytes (Box 1, Glossary), almost 100% of tumors exhibit at least one genetic lesion in a RAS pathway regulator (Schadendorf et al., 2015). For over 40 years, research has been deciphering the molecular mechanisms of RAS regulation, with the goal of translating this knowledge into new treatment strategies that benefit patients with cancer. Recently, tremendous progress in basic science and clinical care has established melanoma as the exemplar of tumors driven by aberrant, but therapeutically targetable, RAS pathway activation. Here, we review the mechanisms of RAS pathway regulation, the genetic basis of RAS pathway dysregulation and the therapeutic approaches that target this pathway by using melanoma as a paradigm.

**Box 1. Glossary**

**Acral melanoma:** a rare subtype of melanoma that develops in the skin of palms and soles (Basurto-Lozada et al., 2021).

**Benign nevus (commonly known as mole):** a common circumscribed skin lesion due to a local proliferation of melanocytes. Benign nevi often harbor *BRAF* mutations but only rarely progress to melanoma.

**Cutaneous melanoma:** an aggressive form of skin cancer that arises from the malignant transformation of skin melanocytes.

**Cytotoxic T-lymphocyte-associated protein 4 (CTLA-4):** an example of an immune checkpoint (Tivol et al., 1995; Waterhouse et al., 1995).

**Immune checkpoints:** pathways that regulate the immune system. They play a crucial role in self-tolerance by inhibiting the cytotoxic activity of T-cells.

**Immunotherapy:** blocks immune checkpoints to reactivate anti-tumor immunity. This can be used against certain cancer cells that activate immune checkpoints to escape immune surveillance.

**Melanocyte:** cell that produces the melanin pigment. Melanocytes are located in the bottom layer of the skin epidermis, as well as in the eye and in epithelial layers lining various internal organs.

**Mucosal melanoma:** arises from melanocytes of mucosal epithelia lining oronasal, anorectal and vulvovaginal cavities (Postow et al., 2012).

**Neurofibromin (NF1):** a major RAS-GAP. The GAP-related domain located in the center of the NF1 protein contains the catalytic site and the RAS-binding region that interacts with RAS switch regions I and II (Scheffzek et al., 1997). NF1 cellular distribution appears mainly cytoplasmic and requires binding to SPRED1 to reach the plasma membrane where active RAS is located (Stowe et al., 2012).

**Programmed cell death protein 1/ programmed cell death 1 ligand 1 (PDCD1/CD274, also known as PD-1/PD-L1):** an example of an immune checkpoint (Dong et al., 1999; Freeman et al., 2000).

**Receptor tyrosine kinases** (**RTKs):** a class of cell-surface receptors for growth factors, cytokines and hormones. They initiate intracellular signaling cascades by phosphorylating one or several substrates upon ligand binding. Examples of RTKs include the stem cell factor receptor KIT, the epidermal growth factor receptor (EGFR), the platelet-derived growth factor receptor (PDGFR) or the insulin-like growth factor 1 receptor (IGF1R).

**SPRED1:** sprouty-related EVH1 domain containing 1, initially discovered as a repressor of RAS signaling (Wakioka et al., 2001). It contains an N-terminal EVH1 domain, a central KIT binding domain and a C-terminal SPROUTY-related domain. The SPROUTY-related domain of SPRED1 is involved in membrane anchorage, whereas the EVH1 domain binds to the GAP-related domain of NF1.

**SPROUTY:** family of proteins that inhibit the RAS/MAPK pathway as a feedback loop, comprising SPRY1, SPRY2, SPRY3, SPRY4. These proteins contain a conserved, C-terminal cysteine-rich region, called the SPROUTY domain that promotes their membrane anchorage.

**Uveal melanoma (also called ocular melanoma):** malignant transformation of melanocytes that are present in the part of the eye known as the uvea. The uvea comprises the iris, ciliary body and choroid (Jager et al., 2020).

## Molecular definition and regulation of the RAS pathway

### Activators and negative regulators of RAS

**Fig. 2.**
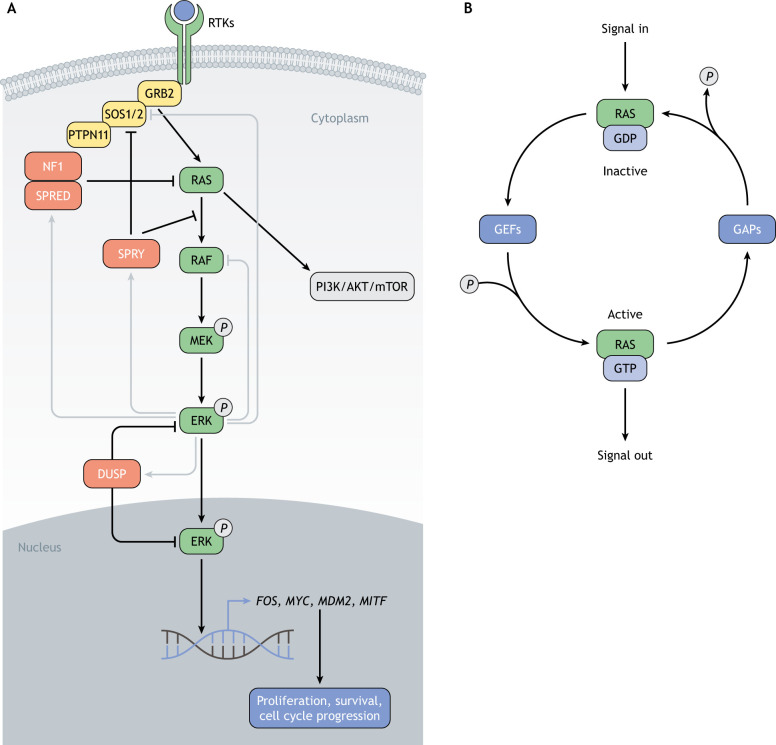
**The RAS pathway.** (A) Growth factor binding to RTKs, such as stem cell factor receptor (KIT), epidermal growth factor receptor (EGFR) or platelet-derived growth factor receptor (PDGFR), leads to receptor autophosphorylation and recruitment of the adaptor protein growth factor receptor bound 2 (GRB2). In turn, GRB2 recruits the guanine exchange factor SOS1/2, activating RAS. A main effector of the RAS pathway is the RAF/MEK/ERK (MAPK) cascade that leads to the transcription of different target genes (e.g. *FOS*, *MYC*, *MDM2*, *MITF*) implicated in cell proliferation, survival and cell cycle progression. The PI3K/AKT/mTOR (PI3K) pathway is another important effector of active RAS, promoting cell growth and implicated in melanoma development. Gray lines represent regulatory feedback loops. These include direct phosphorylation of SOS1/2 and RAFs by ERK, leading to dissociation of GRB2–SOS1/2 and RAS–RAF complexes, respectively, as well as the transcriptional induction of negative regulators, such as members of the DUSP, SPROUTY and SPRED families, i.e. DUSP4, DUSP5, DUSP6, SPRY1, SPRY2, SPRY3, SPRY4, SPRED1 and SPRED2. *P*, phosphate; SPRY, sprouty RTK signaling antagonists 1,2, 3 and 4. (B) Regulation of RAS activity by guanine nucleotide exchange factors (GEFs), such as SOS1 or 2, and GTPase-activating proteins (GAPs), such as NF1. GEFs stimulate the formation of the GTP-bound active state, whereas GAPs catalyze GTP hydrolysis and favor reverting to the GDP-bound inactive state.

The RAS pathway can be activated at the cell membrane by receptor tyrosine kinases (RTKs; Box 1) in response to growth factors (Fig. 2A). Ligand binding stimulates enzymatic activity of the RTK and results in its autophosphorylation (Heldin, 1995), which allows binding to adaptor proteins, such as growth factor receptor-bound protein 2 (GRB2) (Lowenstein et al., 1992). GRB2 is then able to recruit guanine exchange factors (GEFs) (Egan et al., 1993; Li et al., 1993) to catalyze the switch from the inactive GDP-bound form to the active GTP-bound conformation of RAS (Bos et al., 2007) (Fig. 2B). In mammals, the main RAS-GEFs are son of sevenless homolog 1 and 2 (SOS1 and SOS2, respectively) (Bowtell et al., 1992).

Unlike GEFs, GTPase-activating proteins (GAPs) catalyze the hydrolysis of GTP by RAS, as RAS itself has low intrinsic GTPase activity (Hunter et al., 2015). This results in RAS inactivation (Fig. 2B). Neurofibromin 1 (NF1; Box 1) is the first-identified and most-studied RAS-GAP in humans (Ballester et al., 1990; Martin et al., 1990; Xu et al., 1990a,b). Inactivation of NF1 reduces the rate of GTP hydrolysis by RAS to negligible levels, thus increasing the proportion of GTP-bound RAS and leading to the hyperactivation of the pathway (Basu et al., 1992; Bollag et al., 1996; DeClue et al., 1992).

Interestingly, the RAS-inactivating function of NF1 depends on its translocation to the plasma membrane, which is facilitated by sprouty-related EVH1-domain-containing 1 (SPRED1; Box 1) (Stowe et al., 2012), another negative regulator of RAS activity (Wakioka et al., 2001) (Fig. 2A). Recent functional studies and structural data have brought new insights to the molecular regulation of RAS through NF1 and SPRED1, by demonstrating that SPRED1 binds to the GAP-related domain of NF1 without disturbing NF1 binding to RAS (Hirata et al., 2016; Yan et al., 2020). Although several other GAPs have been identified, NF1 appears to be the main direct negative regulator of RAS. Moreover, the variety of RAS regulators underscores the complexity of the RAS pathway regulatory network.

### Downstream effectors and their biological functions

RAS regulates several signaling pathways. The mitogen-activated protein kinase (MAPK) pathway is arguably the most-studied effector of RAS activity. It transduces RAS signals from the cell membrane to the nucleus via a phosphorylation cascade, eventually, recruiting transcription factors to activate specific sets of target genes (Fig. 2A). The first direct target of RAS identified was the serine-threonine-specific protein kinase RAF (Warne et al., 1993; Zhang et al., 1993). There are three homologues of RAF protein: ARAF, BRAF and CRAF (officially known as RAF1). In the presence of growth factors, GTP-bound RAS allosterically activates RAFs (Chuang et al., 1994), while RAFs are simultaneously phosphorylated by other kinases (Fig. 2A). Activated RAFs can then phosphorylate and activate two members of the mitogen-activated protein kinase kinase (MAP2K) family, i.e. MEK1 and MEK2 (officially known as MAP2K1 and MAP2K2, respectively; hereafter referred to as MEK) (Gardner et al., 1994) that, in turn, phosphorylate and activate two members of the mitogen-activated protein kinase (MAPK) family, i.e. ERK1 and ERK2 (officially known as MAPK3 and MAPK1, respectively but, hereafter, referred to as ERK) (Fig. 2A). Active ERK can translocate to the nucleus or phosphorylate its substrates directly in the cytoplasm, of which hundreds have been identified (Ünal et al., 2017) (Fig. 2A). ERK signaling regulates a wide range of biological processes, such as cell cycle progression (Monje et al., 2005) and proliferation (Sears et al., 2000), which signifies the expansive impact of the RAS pathway. In particular, microphthalmia-associated transcription factor (MITF), a master regulator of melanocyte differentiation, represents an important downstream target of the MAPK pathway in the context of melanoma (Wellbrock and Arozarena, 2015), as its transcriptional activity and protein turnover are modulated by ERK or ERK effectors through post-translational modifications, such as phosphorylation, SUMOylation and ubiquitylation (Hemesath et al., 1998; Wu et al., 2000).

Another well-known effect of RAS is exercised via the phosphoinositide 3-kinase (PI3K) pathway (Sjolander et al., 1991) (Fig. 2A). PI3Ks are a family of lipid kinases and are activated by GTP-bound RAS, initiating a series of phosphorylation events that regulate several signaling proteins, including the serine-threonine protein kinases AKT1, 2 and 3 (hereafter referred to as AKT) (Fig. 2A). AKT phosphorylates many different substrates (Alessi et al., 1996), leading, in particular, to degradation of TP53 (Zhou et al., 2001) and the inhibition of various pro-apoptotic proteins (Brunet et al., 1999), thus blocking apoptosis. In addition, AKT triggers a cascade of events resulting in the activation of the mechanistic target of rapamycin (mTOR), a major regulatory hub for translation and growth (Chung et al., 1992; Navé et al., 1999) (Fig. 2A). Interestingly, the phosphatase and tensin homolog (PTEN), which represses a phosphorylation step downstream of PI3Ks, is a major melanoma tumor suppressor (Li et al., 1997). Many other effectors of RAS activation have been reported (Khan et al., 2019); however, by focusing on the MAPK and PI3K pathways alone, it is evident that RAS controls multiple cellular processes, including cell proliferation and growth, cell survival and apoptosis as well as angiogenesis and cell migration, dysregulation of which can lead to malignant transformation.

### Regulatory feedback loops

Owing to its crucial importance in essential cellular processes, the RAS pathway is tightly regulated. Multiple feedback loops fine-tune its level of activation (Fig. 2A). It was first noticed that activation of the RAS pathway upon growth factor receptor stimulation is transient despite continuous presence of a ligand. Deactivation of the pathway has been linked to a negative feedback loop, involving phosphorylation of SOS1 and SOS2 by ERK and subsequent dissociation of the GRB2–SOS1/2 complex (Cherniack et al., 1995; Waters et al., 1995). Direct phosphorylation of RAF by ERK has also been proposed to disrupt the RAS/RAF interaction, thus inhibiting further pathway activation (Dougherty et al., 2005) (Fig. 2A).

In addition, several direct RAS regulators are transcriptionally controlled through the MAPK pathway itself. Indeed, a study investigating the immediate changes in gene expression upon MEK inhibition within melanoma cell lines with hyperactive MAPK signaling, identified several members of the dual specificity phosphatase (DUSP), sprouty RTK-signaling antagonist (SPROUTY; Box 1) and sprouty-related EVH1 domain containing (SPRED) families, namely *DUSP4*, *DUSP6*, *SPRY2*, *SPRY4* and *SPRED2*, as drastically downregulated (Pratilas et al., 2009) (Fig. 2A). DUSPs comprise a large family of phosphatases that dephosphorylate phosphorylated tyrosine (Y), serine (S) or threonine (T) residues. A subset of them is involved in the MAPK pathway. DUSP5 and DUSP6, for example, dephosphorylate ERK in the nucleus and the cytoplasm, respectively, thus terminating the pathway (Karlsson et al., 2004; Kidger et al., 2017). SPROUTYs have initially been identified in *Drosophila* as repressors of FGF signaling (Hacohen et al., 1998) and, later, as negative regulators of the RAS pathway, preventing the activation of the GRB2–SOS1/2 complex and inhibiting RAS-induced activation of RAFs (Hanafusa et al., 2002; Yusoff et al., 2002). In addition to the transcriptional upregulation of SPROUTY family members by RAS pathway effectors, their RTK-induced phosphorylation seems crucial for their binding to GRB2, suggesting the existence of multiple RAS-mediated ways to activate SPROUTY proteins. Finally, SPRED proteins facilitate NF1-mediated inhibition of RAS. In human melanoma cell lines, SPRED1 levels are modulated via the RAS pathway, suggestive of a negative feedback loop similar to that regarding SPROUTY proteins (Ablain et al., 2021). All these feedback mechanisms form a network that plays a crucial role in controlling the intensity and duration of RAS pathway signaling, and their importance in cell homeostasis is highlighted by their frequent inactivation in cancer (Courtois-Cox et al., 2006).

### Regulation of cellular localization

**Fig. 3.**
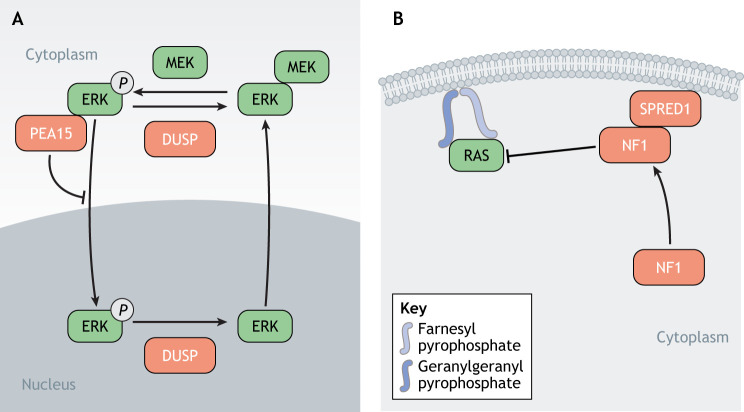
**The RAS pathway – regulation of its cellular localization.** (A) MEKs contain a nuclear export signal that keeps the MEK–ERK complex within the cytoplasm if the RAS/MAPK pathway has not been activated. Upon pathway activation, ERK is phosphorylated by MEK, which leads to the dissociation of the complex, allowing ERK to translocate to the nucleus and to interact with substrates. The scaffold protein PEA15 can also sequester ERK in the cytoplasm by preventing its interaction with nucleoporins. Dephosphorylation of nuclear ERK by DUSPs mediates its translocation back to the cytoplasm. (B) RAS requires membrane localization to be active. Addition of a farnesyl or a geranylgeranyl group to its hypervariable region (HVR) by farnesyl transferase or geranylgeranyl prenyltransferase, respectively, facilitates RAS membrane anchorage. In order to implement its RAS-GAP activity and to downregulate RAS, NF1 requires interaction with SPRED1 and localization to the membrane, thereby achieving physical proximity with RAS.

In addition to protein–protein interactions, the cellular localization of RAS pathway members modulates the activity of the pathway and influences its output (Fig. 3). For instance, the shuttling of phosphorylated ERK in and out of the nucleus is tightly controlled. MEK1 and MEK2 contain a nuclear export signal that keeps the MEK/ERK complex in the cytoplasm in the absence of RAS/MAPK pathway stimulation (Fukuda et al., 1997a) (Fig. 3A). Upon pathway activation, MEK phosphorylates ERK, which dissociates the MEK–ERK complex, then allowing ERK to interact with nuclear pores and reach the nucleus (Matsubayashi et al., 2001; Whitehurst et al., 2002) (Fig. 3A). After inactivation of the pathway, the export of dephosphorylated ERK from the nucleus also seems to depend on MEK (Fukuda et al., 1997b). Another proposed mechanism involves the scaffold protein PEA15 that sequesters ERK in the cytoplasm by preventing its interaction with nucleoporins, thus modulating the phosphorylation of ERK substrates and interfering with downstream signaling (Formstecher et al., 2001; Whitehurst et al., 2004) (Fig. 3A).

Another illustration of the importance of cellular localization in the regulation of RAS pathway activity is the membrane tethering of RAS and its direct interactors. In order to be active, RAS proteins need anchorage to the membrane, which requires post-translational modifications of the short HVR in their C-terminus (Willumsen et al., 1984). One example is isoprenylation, an irreversible lipidic modification that consists of the addition of a fatty acid chain, such as a farnesyl or a geranylgeranyl group (Hancock et al., 1989) (Fig. 3B). This is performed by either farnesyl transferase or geranylgeranyl prenyltransferase (Zhang and Casey, 1996) (Fig. 3B).

Interestingly, activity of NF1 also depends on its membrane localization. NF1 does not contain any membrane-associated domain and, therefore, relies on its interaction with SPRED1 to localize to the membrane and achieve physical proximity with RAS (Fig. 3B). Indeed, the cysteine-rich SPROUTY-related domain of SPRED1, which is responsible for its membrane association (Lim et al., 2002), is also required for NF1 targeting to the plasma membrane and for NF1-mediated repression of RAS activity (Stowe et al., 2012). Membrane tethering of the RAS–NF1–SPRED1 complex, the structure of which was recently resolved (Yan et al., 2020), is thus emerging as a key mechanism for pathway regulation. The local concentrations of RAS, GEFs and GAPs at the plasma membrane may, indeed, facilitate guanine exchange and switch of RAS activity, thus increasing the responsiveness of the pathway to input signals.

## Clinical manifestation of RAS pathway alterations

### RAS mutations in cancer

**Fig. 4.**
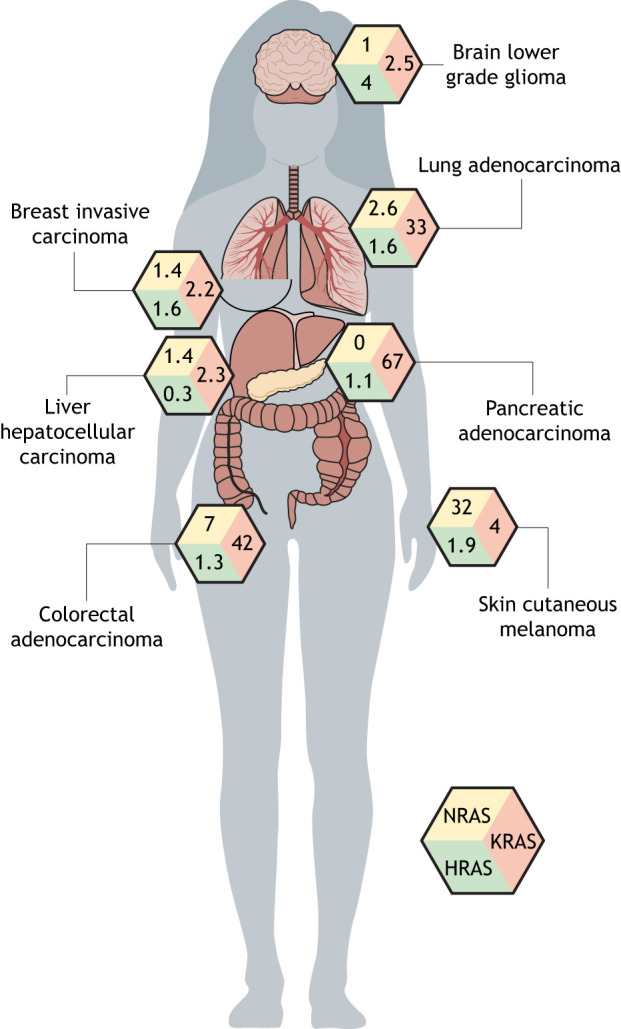
**Frequency of RAS isoform (HRAS, NRAS, KRAS) mutations in human cancers.** The proportion of human tumors with mutations in each RAS gene is indicated for some of the most-common types of cancer, demonstrating the prevalence of RAS mutations in driving cancer and underscoring the need for therapeutic strategies that specifically target the RAS pathway.

RAS genes are mutated in close to a third of all human cancers (Hobbs et al., 2016) and, in some tumor types, they even represent the main oncogenic driver (Fig. 4). For example, activating mutations in *KRAS* have been reported in 60–90% of pancreatic cancer cases (Bailey et al., 2016; Zehir et al., 2017), whereas in colorectal and lung cancers, they are fond in 30–50% of tumors (Campbell et al., 2016; Yaeger et al., 2018). *NRAS* is mutated in ∼30% of melanomas (Akbani et al., 2015; Hodis et al., 2012).

KRAS is most frequently altered on glycine at position 12 (Fig. 1), which prevents proper interaction with GAPs and, thus, inhibits GTP hydrolysis and inactivation (Scheffzek et al., 1997). Although the three RAS genes are extremely similar in both sequence and protein structure – and despite them sharing oncogenic properties – their functions, as assessed in knockout mouse models, do not entirely overlap (Table 1). For example, mutant *NRAS* failed to recapitulate the oncogenic effect of mutant *KRAS* in mouse colonic epithelium (Haigis et al., 2008). Furthermore, *KRAS* is the most-expressed RAS gene in mouse tissues (Newlaczyl et al., 2017) and the only one that is embryonic lethal when knocked out (Koera et al., 1997) (Table 1). Although the exact reasons for the apparent tissue specificity of the different RAS oncogenes remain obscure, these observations suggest that *KRAS* is the functionally dominant RAS gene in many tissues and might explain why it is the most frequently mutated in human cancers. It is likely that future therapeutic strategies against RAS-driven tumors will need to be adapted to the specificities and even the type of mutations of each RAS gene.

**Table 1.**
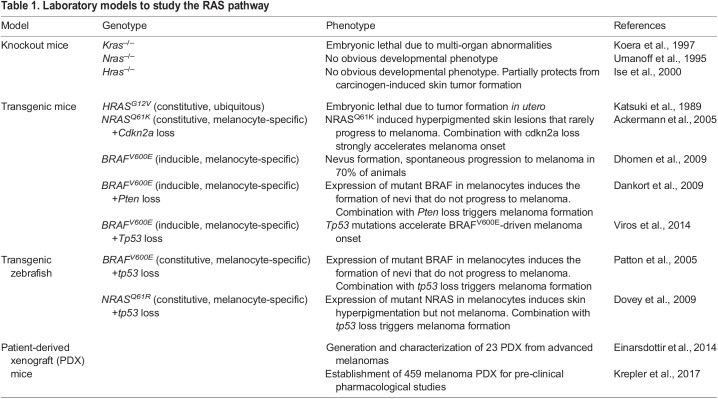
Laboratory models to study the RAS pathway

### RAS pathway alterations in cutaneous melanoma

Melanoma is a disease of aberrant RAS pathway activation. Virtually all human melanomas exhibit at least one genetic lesion causing RAS pathway hyperactivation that drives oncogenic transformation and sustains cancer growth (Cohen et al., 2002). It is important to distinguish between the different melanoma subtypes, as their driving genetic alterations differ (Hayward et al., 2017). In cutaneous melanoma (Box 1), the most frequently mutated driver gene is the RAS effector *BRAF* (Berger et al., 2012; Hodis et al., 2012; Krauthammer et al., 2012), which is altered in 50% of cases (Table 2). Class I BRAF mutations, affecting valine (V) at position 600, account for 80% of alterations and confer constitutive kinase activity to the BRAF monomer, which becomes independent of upstream RAS-signaling (Davies et al., 2002; Wan et al., 2004). Other mutations that affect different residues of BRAF protein were categorized by the group of Neil Rosen as class II mutants allowing BRAF to signal as a dimer independently of RAS activity (Yao et al., 2015), and class III mutants that display low kinase activity but bind to RAS and CRAF more tightly and, thus, amplify RAS downstream signaling (Heidorn et al., 2010; Wan et al., 2004; Yao et al., 2017). Rare gene fusions implicating the *BRAF* gene have also been reported (Hayward et al., 2017; Palanisamy et al., 2010). Interestingly, *BRAF* mutations have also been detected in other tumor types including colorectal and brain cancers (Davies et al., 2002). The prevalence of BRAS mutations at V600 in melanoma has prompted the development of inhibitors specifically directed against these mutants (Bollag et al., 2010), but small molecules targeting other mutant classes have also recently been identified (Yao et al., 2019).

**Table 2.**
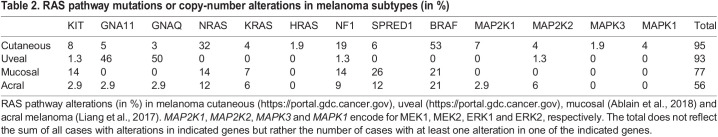
RAS pathway mutations or copy-number alterations in melanoma subtypes (in %)

NRAS is mutated in ∼30% of cutaneous melanomas (Akbani et al., 2015) (Fig. 4), mostly at the Q61 residue in the switch II region (Fig. 1) that abolishes RAS GTPase activity and, thus, results in constitutive activation. Mutations in the other RAS genes *KRAS* and *HRAS* are much rarer, occurring in 3% and 2% of cutaneous melanoma, respectively.

The third major oncogenic lesion in melanoma affects the tumor suppressor NF1. Loss-of-function alterations of *NF1* are present in almost 20% of melanomas and include missense or truncating mutations as well as chromosomal deletions (Akbani et al., 2015; Krauthammer et al., 2015). These lesions abolish the RAS-GAP catalytic activity of NF1, leading to constitutive RAS activation. Importantly, another RAS-GAP, Ras GTPase-activating protein 2 (RASA2), is inactivated in a significant proportion of melanoma (Arafeh et al., 2015). Finally, chromosomal amplifications or activating mutations of the receptor tyrosine kinase *KIT* are found in 5–10% of cutaneous melanomas and confer independence from external growth factors, thus representing the most-upstream source of RAS pathway hyperactivation.

Overall, alterations in *BRAF*, *NRAS*, *NF1* and *KIT* are present in 93% of human cutaneous melanomas in a mutually exclusive pattern, although a small fraction of tumors harbor alterations in several of these genes (Akbani et al., 2015; Hayward et al., 2017; Hodis et al., 2012) (Table 2). These data reinforce the idea that activation of the RAS/MAPK pathway constitutes the driving oncogenic force in this tumor type. This idea is also supported by strong experimental evidence in genetically engineered animal models that recapitulate *in vivo* the genetics of human melanoma (Table 1). RAS pathway regulators are highly conserved between vertebrate species and, in these models, expression of mutant *BRAF* or *NRAS* under the control of a melanocyte-specific promoter induces the formation of benign nevi (Box 1), as it does in humans. However, these mutations only initiate melanoma when combined with inactivation of a tumor suppressor gene, such as *CDKN2A*, *PTEN* or *TP53* (Ackermann et al., 2005; Dankort et al., 2009; Dhomen et al., 2009; Dovey et al., 2009; Patton et al., 2005). A genomic classification of cutaneous melanoma was proposed on the basis of these observations, upon the release of sequencing data from hundreds of human melanoma samples by The Cancer Genome Atlas (TCGA) consortium, and four groups were thus defined, i.e. *BRAF*-mutant, *NRAS*-mutant, *NF1*-mutant, and triple wild-type (WT) group (Akbani et al., 2015). The latter represents <10% of melanomas and includes *KIT*-driven tumors. It is likely that triple-WT tumors also rely on hyperactive RAS/MAPK, but their drivers are less-common members of this pathway, not known to play a role in cancer or might be RAS pathway regulators that have not yet been discovered. Indeed, mutations in other RAS pathway genes, such as the upstream regulators *SOS1*, *SOS2*, *GRB2* and *PTPN11* (also known as SHP2) and in genes encoding downstream effectors MEK1/2 and ERK1/2, can be detected in ≤5% of cutaneous melanomas. A more-complete characterization of these minor alterations would not only expand our knowledge of RAS pathway regulation but might also reveal new opportunities for innovative treatment strategies against the non-BRAF mutant genetic subtypes of melanoma, for which there are currently no targeted therapy options.

### RAS pathway alterations in rare melanoma subtypes

The proportions of genetic alterations in members of the RAS pathway vary between cutaneous melanoma and rare melanoma subtypes, such as uveal, mucosal and acral melanoma (Box 1), which account for a small percentage of all melanomas (Alicea and Rebecca, 2021) (Table 2). Uveal melanoma (Box 1) has radically different genetic causes compared to cutaneous melanoma because mutations in *BRAF* or *NRAS* are exceedingly rare. Instead, >90% of uveal melanoma harbor oncogenic driver mutations in *GNA11* or *GNAQ*, two genes encoding G proteins that can directly activate the MAPK pathway (Johansson et al., 2020; Van Raamsdonk et al., 2009) (Table 2).

As mucosal and acral melanoma arise from areas of the body that are rarely exposed to sunlight, their mechanisms of tumorigenesis are different from those leading to cutaneous melanoma. Therefore, the genetic landscape of these tumors is characterized by a lower mutation burden and the absence of UV-induced DNA-damage signatures. However, mucosal and acral melanoma display more copy-number alterations and complex chromosomal rearrangements compared to cutaneous melanoma (Hayward et al., 2017). *BRAF* mutations are less common in these subtypes – apart from class III mutants that potentiate tighter binding to RAS and CRAF, which are more prevalent in acral and mucosal melanoma (Box 1) than in cutaneous melanoma. Interestingly, alterations in upstream regulators of the RAS pathway, such as activation of KIT, are also more prevalent (Ablain et al., 2018; Liang et al., 2017; Newell et al., 2019, 2020) (Table 2). Our own group recently discovered that the negative regulator of RAS, *SPRED1*, is a major tumor-suppressor gene deleted in >26% of mucosal melanomas (Ablain et al., 2018). Although mutations in *SPRED1* and *KIT* are relatively weaker activators of the RAS pathway compared to the *BRAF^V600E^* or *NRAS^Q61R^* mutants, they frequently occur together to drive mucosal melanoma (Ablain et al., 2018). Since neither *BRAF^V600E^* nor *NRAS^Q61R^* are induced by UV light, it is still unclear whether the genetic differences between melanoma subtypes reflect different modes of mutational acquisition or depend on the nature and/or environment of the melanocyte population. Owing to the relative rarity of some of these subtypes, the genetic landscapes of acral and, to a lesser extent, mucosal melanoma are still incompletely defined. However, recently launched initiatives will provide a more-precise and thorough description of RAS pathway alterations in these tumors, with the possibility of unconventional RAS pathway regulators, perhaps those associated with developmental disorders rather than cancer (see Box 2), being identified as new oncogenic drivers.

**Box 2. RASopathies inform RAS pathway function and regulation**

In addition to its frequent involvement in cancer, genetic dysregulation of the RAS pathway causes several developmental disorders called RASopathies that, together, affect ∼1 in 1000 individuals (Rauen, 2013). The identification of the causative germline mutations has established a network of crucial RAS regulators. Moreover, these rare syndromes share several clinical manifestations that have informed the biological functions as well as lineage specificity of RAS pathway regulators. RASopathies include Noonan syndrome (Pandit et al., 2007; Tartaglia et al., 2001), Noonan syndrome with multiple lentigines (Digilio et al., 2002; Pandit et al., 2007), neurofibromatosis type 1 (Viskochil et al., 1990; Wallace et al., 1990), Legius syndrome (Brems et al., 2007), capillary malformation–arteriovenous malformation syndrome (Eerola et al., 2003), Costello syndrome (Aoki et al., 2005) and cardio-facio-cutaneous syndrome (Rodriguez-Viciana et al., 2006), each of which is characterized by a unique set of mutations (Table 3). As in melanoma, these genetic lesions act at different levels of the RAS pathway and all lead to its aberrant activation.

Noonan syndrome and the rare autosomal-dominant disorder Noonan syndrome with multiple lentigines (formerly known as LEOPARD syndrome) are characterized by congenital heart defects, short stature and developmental delay of variable degree. They are linked to over 18 genes principally encoding upstream positive regulators of the RAS pathway. The most-common mutations are gain-of-function mutations affecting the RAS activator *PTPN11* (Tartaglia et al., 2001). Several mechanisms of action have been proposed for the PTPN11 phosphatase (Dance et al., 2008), including activation of SPROUTY proteins (Hanafusa et al., 2004), activation of SRC family kinases (Cunnick et al., 2002) or even direct dephosphorylation of RAS (Bunda et al., 2015). The second most-frequent genetic lesions found in patients with Noonan syndrome are mutations in the RAS-GEF *SOS1*, which abrogate the SOS1 autoinhibitory function and result in its constitutive activation (Roberts et al., 2007; Tartaglia et al., 2007).

Neurofibromatosis type 1, Legius syndrome and capillary malformation–arteriovenous malformation syndrome are all associated with the inactivation of direct negative regulators of RAS. Neurofibromatosis type 1 is the most-common and best-known RASopathy (Williams et al., 2009). It is caused by mutations in the *NF1* gene resulting in loss-of-function, which is also the case in cutaneous melanoma (Krauthammer et al., 2015). The disease manifests as café-au-lait spots on the skin and as tumors, such as gliomas and neurofibromas (Shen et al., 1996). Legius syndrome (also called NF1-like syndrome) is only differentiated from neurofibromatosis type 1 by the absence of non-pigmentary clinical manifestations, such as tumors (Brems et al., 2007). It is caused by heterozygous inactivating mutations in the gene encoding the other major negative regulator of RAS, *SPRED1*, which have also been implicated in mucosal melanoma (Ablain et al., 2018). The clinical similarities between neurofibromatosis type 1 and Legius syndrome reflect the inter-dependent functions of the two causative genes in RAS regulation. Indeed, structural and functional studies demonstrated that the mutations found in the two syndromes disrupt the interaction between NF1 and SPRED1 (Dunzendorfer-Matt et al., 2016; Hirata et al., 2016). Capillary malformation–arteriovenous malformation syndrome is also due to the inhibition of a RAS-GAP. Heterozygous inactivating mutations in *RASA1* have been linked to this disorder of the vascular system, characterized by capillary and cardiovascular malformations (Eerola et al., 2003).

Finally, Costello syndrome and cardio-facio-cutaneous syndrome are usually diagnosed through cardiac anomalies and caused by activation of the RAS pathway at the level of RAS itself or of its downstream effectors. Costello syndrome is due to heterozygous activating mutations in *HRAS* (Aoki et al., 2005), whereas cardio-facio-cutaneous syndrome is caused by mutations in *KRAS*, *BRAF* or *MEK1/2* (Rodriguez-Viciana et al., 2006).

RAS signaling is essential to many cell functions in most tissues, both during development and at homeostasis. Hence, it is not surprising that inherited dysregulation of the RAS pathway should elicit systemic manifestations. Yet, RASopathies exhibit common phenotypic features that include cutaneous lesions, craniofacial abnormalities, cardiac malformations, neurological dysfunction and increased cancer risk. This observation points to a common mechanism behind the various mutations identified in these syndromes and, possibly, also to specific cell types affected by this mechanism. In this respect, it is interesting to note that the common manifestations of RASopathies, such as pigmentation abnormalities, can be linked to defects in cell types derived from the neural crest (the embryonic cell lineage that gives rise to melanocytes), further highlighting the parallel between RASopathies and melanoma. It remains to be elucidated whether the clinical differences between the various RASopathies reflect differences in the way the RAS pathway is activated or, rather, variations in the sensitivity of different cell lineages to specific mutations. The study of the molecular and cellular manifestations of RASopathies could provide detailed understanding of the function of each RAS pathway regulator in a context devoid of additional genetic alterations and might, thus, cast new light on the functional consequences of their mutation in melanoma.

**Table 3.**
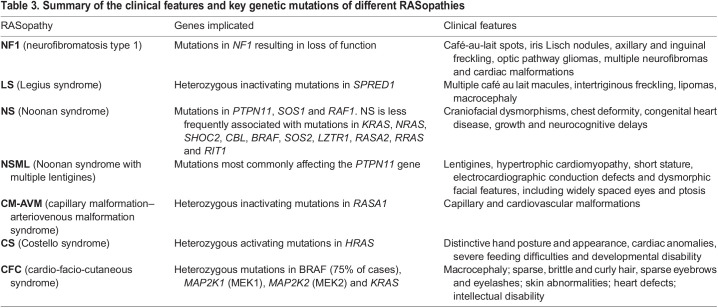
Summary of the clinical features and key genetic mutations of different RASopathies

## Treating RAS pathway-driven cancer

In the past 15 years, the prognosis of patients with melanoma has radically changed. In the early 2000s, although localized lesions could be surgically removed with a cure rate close to 100%, metastatic disease was associated with extremely poor outcomes, a median survival of 6 months after diagnosis and a 5-year overall survival of under 5% (Korn et al., 2008). The development of therapies targeting the MAPK pathway, followed by the introduction of immunotherapies has revolutionized the clinical management of patients with metastatic melanoma (Schadendorf et al., 2018).

### Therapies targeting the RAS pathway

As the development of melanomas heavily relies on RAS pathway mutations, an array of drugs targeting this pathway has been developed and is used in the clinic with varying degrees of success. Based on the mutational landscape of human cutaneous melanoma and on protein structure data, drugs were designed to specifically block the most-common BRAF mutant, BRAF^V600E^ (Bollag et al., 2010). Vemurafenib and, later, dabrafenib, elicited partial responses in most patients with metastatic *BRAF*-mutant melanoma, and some patients presented complete responses with impressive tumor shrinkage (Flaherty et al., 2010; Hauschild et al., 2012). Treatment improved overall survival at 6 months by 30% compared to standard chemotherapy, providing an average benefit of over 3 months (Chapman et al., 2011). However, tumors invariably developed resistance to BRAF inhibition (Sosman et al., 2012). To augment the arsenal of targeted therapies against the RAS/MAPK pathway and delay drug resistance, the MEK inhibitors trametinib and cobimetinib were rapidly tested. Although they only showed modest clinical efficacy as single agents (Flaherty et al., 2012a), they had a significant anti-cancer impact when combined with BRAF inhibitors. The drug combination significantly improved response rates, progression-free survival and overall survival compared to single-agent BRAF inhibition (Flaherty et al., 2012b; Larkin et al., 2014; Long et al., 2014; Robert et al., 2015a). Five-year outcomes after combination therapy showed a complete response rate of 19%, a median overall survival of 26 months and a 5-year overall survival rate of 34% in patients with metastatic melanoma, who, historically, had extremely poor prognosis (Robert et al., 2019).

Targeted therapy options are scarcer for patients with non-*BRAF*-mutated melanoma. No direct inhibitors of NRAS are available and initial studies suggested lower sensitivity of *NRAS*-driven tumors to MEK inhibition compared to *BRAF*-driven ones *in vitro* and in preclinical xenograft models (Solit et al., 2006). However, the second-generation MEK inhibitor binimetinib elicited similar rates of partial response, i.e. ∼20% in patients with *NRAS*- and *BRAF*-driven melanoma in a phase II clinical trial (Ascierto et al., 2013). The reported median progression-free survival under binimetinib treatment was 2.8 months compared to 1.5 months under standard chemotherapy in patients with *NRAS*-mutant melanoma (Dummer et al., 2017). Despite the partial responses and a modest improvement in disease evolution, the impact of MEK inhibition alone appears limited in these patients. This limited clinical efficacy may be due to insufficient target inhibition, which might be overcome by combining MEK and ERK inhibitors, a treatment modality that induces better responses than either agent alone in *NRAS*-mutant melanoma *in vitro* (Rebecca et al., 2014). The efficacy of MEK inhibitors may also be hindered by treatment-related toxicities and/or the protective effect of signaling pathways other than the MAPK pathway, such as PI3K/AKT, that are active downstream of mutant RAS.

Little is known about the response of *NF1*-driven melanoma to targeted therapies. Based on the molecular mode of action of NF1, one could predict transient and partial sensitivity to MEK inhibition, a hypothesis supported by *in vitro* data (Nissan et al., 2014). More studies are needed to identify vulnerabilities, potential drug targets and new treatment strategies in this genetic subtype. In contrast, melanomas with WT *BRAF*, *NRAS* and *NF1* that are driven by activating *KIT* mutations can be treated with the RTK inhibitor imatinib, yielding response rates of 16–30% (Carvajal et al., 2011; Guo et al., 2011; Hodi et al., 2013); however, most of these responses were partial and transient. Later trials using the more-specific KIT inhibitor nilotinib showed similar responses (Carvajal et al., 2015; Delyon et al., 2018; Guo et al., 2017; Lee et al., 2015). This therapeutic strategy, although successful, suffers from the same limitations as MEK inhibition in *NRAS*-driven tumors. However, combining KIT and MEK inhibitors may represent a valuable treatment option for patients with KIT-mutant melanoma. Addition of PI3K/AKT inhibitors to the treatment regimen could also be explored as KIT-driven tumors seem to rely on the activity of this parallel pathway for growth and survival (Todd et al., 2014). Promising preclinical data suggest that strategies combining MAPK and PI3K inhibitors may similarly prove beneficial in *NRAS*- and *NF1*-driven melanoma (Petit et al., 2019; Posch et al., 2013).

### Immunotherapies and RAS pathway alterations

Of all solid cancers, melanomas have the highest rate of response to immunotherapy (Box 1) (Waldman et al., 2020). This might, at least partially, be due to the high number of neoantigens produced by melanoma cells, which facilitates recognition of tumor cells by immune cells. Indeed, melanoma is the tumor type that exhibits the highest mutational burden of all cancers (Alexandrov et al., 2013), and mutation and neoantigen loads appear significantly correlated with the response to immunotherapy and clinical benefit (McGranahan et al., 2016; Snyder et al., 2014; Van Allen et al., 2015). It is noteworthy, however, that this correlation is rather weak, and that mutation load does not always predict treatment outcome, suggesting the existence of other predictive factors. Antibodies against the immune checkpoints CTLA-4 or PDCD1/CD274 (also known as PD-1/PD-L1) (Box 1) can reactivate the T-cell anti-tumor activity that is abrogated in many cancers (Leach et al., 1996; Melero et al., 2007). The combination of CTLA-4 and PD-1 blockade in patients with advanced melanoma has demonstrated an unprecedented response rate of 58%, translating into a median overall survival rate of >5 years, and a 5-year overall survival rate of 52% (Larkin et al., 2015a, 2019; Postow et al., 2015), making this drug combination the standard of care in unresectable melanoma.

After early suggestions that non-*BRAF*-driven melanomas exhibit different sensitivities to immunotherapy, subsequent studies concluded that all genetic subtypes similarly respond to immune checkpoint blockade. For instance, nivolumab – an antibody against PD-1 – induced objective responses in 40% of patients with *BRAF*-WT metastatic melanoma (Robert et al., 2015b), which was similar to response rates in cohorts where genetic alterations were not an inclusion criterion (Larkin et al., 2019). A retrospective analysis of several clinical trials, indeed, concluded that nivolumab has comparable efficacy in patients with WT or mutant *BRAF* melanoma (Larkin et al., 2015b). Other retrospective studies focusing specifically on *NRAS*-driven tumors, however, produced conflicting results. One study reported a significantly higher response rate and clinical benefit for patients with *NRAS*-mutant melanoma compared to patients with *NRAS*-WT tumors receiving immunotherapy (Johnson et al., 2015a). In this study, the difference observed under inhibition of PD-1 was attributed to higher levels of PD-L1 detected in *NRAS*-mutant tumors, even though previous reports had found no significant differences in PD-L1 expression in human melanoma cell lines of different genotypes (Atefi et al., 2014). In a larger cohort, however, patients with *NRAS*-mutant tumors showed comparable rates of response to immunotherapy but significantly shorter overall survival compared to patients with *NRAS*-WT melanoma (Kirchberger et al., 2018). Interestingly, in patients with *NRAS*-mutant melanoma, MEK inhibition in combination with immunotherapy tended to improve survival, but prospective studies are warranted to confirm this.

The idea that *NRAS*-driven melanoma might be more sensitive to immunotherapy may relate to the slightly higher average number of mutations in these tumors compared to *BRAF*-driven tumors (Akbani et al., 2015; Krauthammer et al., 2012). Importantly, a much greater difference in mutation load exists between other melanoma genetic subtypes: on average, *NF1*-driven tumors display the highest and triple-WT tumors the lowest mutation rates of all four subtypes (Akbani et al., 2015). Whether these differences are associated with distinct sensitivities to immune checkpoint blockade remains to be determined. In this regard, patients with mucosal melanoma that, generally, belongs to the triple-WT subgroup and exhibits low mutation rates due to the absence of UV-induced DNA damage, benefit less from immunotherapy compared with patients with cutaneous melanoma (D'Angelo et al., 2017; Hamid et al., 2018; Shoushtari et al., 2016). For example, their response rate to single-agent PD-1 blockade is 19–23%, whereas it reaches 40% in patients with cutaneous tumors. Interestingly, among patients with mucosal melanoma, therapy response did not correlate with mutation load (Buchbinder et al., 2021). There remains much to understand about the relationship between tumor genotype and response to immunotherapy in melanoma, in order to develop more-efficient treatment regimens that combine immunotherapy and targeted therapy, especially in melanoma subtypes with low response rates.

### RAS pathway regulators in drug sensitivity or resistance

Despite the remarkable anti-tumor efficacy demonstrated by BRAF inhibitors in the clinic, the development of drug resistance considerably limits long-term survival benefits. The identification of mechanisms of resistance to targeted therapies in melanoma, thus, became the focus of intense investigation – especially by the groups of Roger Lo and Levi Garraway – in the hope that interfering with these mechanisms would lead to the development of improved therapeutic approaches. Two main strategies were used: the *in vitro* generation and characterization of drug-resistant cell lines, and the comparison of the genetic landscape of sensitive and resistant tumors.

The generation of BRAF-inhibitor-resistant cells by pharmacologic exposure *in vitro* and their analysis identified *NRAS* mutations, *MAP3K8* overexpression and the activation of RTKs, such as platelet-derived growth factor receptor beta (PDGFRB), insulin-like growth factor 1 receptor (IGF1R) or MET proto-oncogene, receptor tyrosine kinase (MET), as possible resistance mechanisms through their joint stimulation of the MAPK and PI3K pathways (Johannessen et al., 2010; Nazarian et al., 2010; Straussman et al., 2012; Villanueva et al., 2010). Consistent with a key role of RAS in the orchestration of drug resistance in melanoma, *NF1* was the top hit in a large-scale short hairpin RNA (shRNA) screen for genes that, when lost, confer resistance to BRAF inhibition in melanoma cell lines (Whittaker et al., 2013). The role of *NF1* loss-of-function in the reactivation of the RAS/MAPK pathway upon BRAF inhibition was confirmed in multiple other melanoma cell lines and in an *Nf1*-knockout mouse model (Maertens et al., 2013; Nissan et al., 2014). Interestingly, cells with *NF1* loss-of-function retained sensitivity to MEK or WT RAF inhibition, suggesting that treatment regimens including a MEK inhibitor suppress this resistance mechanism, and further supporting the use of combination therapies with MEK inhibitors in the clinic. In line with these observations, a recent study from our group implicated *SPRED1* deletions in the resistance to targeted therapies in *BRAF*-driven melanoma, demonstrating that loss of *SPRED1* sustains the activity of WT RAS and reactivates the MAPK pathway in the context of BRAF-inhibition *in vitro* and *in vivo* (Ablain et al., 2021).

Sequencing studies revealed genomic amplifications of mutant *BRAF* (Shi et al., 2012), expression of *BRAF* splicing variants (Poulikakos et al., 2010) or acquisition of mutations within *MEK1* or *MEK2* (Van Allen et al., 2014; Wagle et al., 2011, 2014) as frequent drivers of resistance to BRAF inhibition via the reactivation of the RAS pathway in melanoma patients. Interestingly, additional activating mutations in *BRAF* that circumvent pharmacologic inhibition were not observed, contrasting with observations on targeted therapies in other cancers and suggesting that additional *BRAF* mutations are either harder to acquire under treatment pressure or unable to prevent inhibitor binding. Later analyses of genetic data from multiple clinical trials of BRAF inhibitors in melanoma found putative resistance-causing mutations in 58–74% of progressive tumors and confirmed the reactivation of the RAS/MAPK pathway itself as the major cause of resistance to targeted therapy (Johnson et al., 2015b; Shi et al., 2014; Van Allen et al., 2014). Between 63 and 89% of these alterations affected the RAS pathway through *NRAS* or *KRAS* mutations (in 25–41% of cases), *BRAF* splice variants (22–28%), *BRAF* amplifications (13–32%) or *MEK1/2* mutations (4–34%) (Johnson et al., 2015b; Shi et al., 2014; Van Allen et al., 2014). Tumors without MAPK pathway mutations harbored alterations in the PI3K/AKT pathway, including mutations or deletions of *PTEN*, mutations of AKT genes, mutations of *PIK3CA* or *PIK3R1*, or amplifications of genes encoding RTKs (Johnson et al., 2015b; Shi et al., 2014; Van Allen et al., 2014). Although MEK and AKT inhibitors should overcome these resistance mechanisms, some of the alterations mentioned above were found in the context of BRAF/MEK dual inhibition, indicating that, in some modalities, targeting downstream pathways is not sufficient to halt tumor growth. In this respect, detailed genomic analysis of melanomas with acquired resistance to inhibition of both BRAF and MEK demonstrated amplification of the changes detected in tumors resistant to single-agent therapy as well as more-frequent combinations of genetic alterations that affect both MAPK and PI3K pathways (Moriceau et al., 2015). These results suggest a model whereby the accumulation of sensitivity-reducing events confers partial drug tolerance that eventually results in full therapy resistance.

Over the past few years, non-genomic modes of resistance have emerged. Broad transcriptional changes and the expression of specific gene signatures have been implicated in acquired resistance to targeted therapy (Hugo et al., 2015; Johannessen et al., 2013). The ability of cancer cells to transition between different transcriptional states, i.e. plasticity, allows them to adapt to environmental conditions, in particular to drug exposure (Arozarena and Wellbrock, 2019). Four states spanning the melanocyte differentiation trajectory were recently defined in melanoma – the undifferentiated, neural crest-like, transitory and melanocytic state (Tsoi et al., 2018). Sensitivity to targeted therapy appears to correlate with cell differentiation, the more proliferative melanocytic state being sensitive to MAPK inhibitors, and the more migratory undifferentiated state being associated with drug resistance (Konieczkowski et al., 2014). Single-cell RNA sequencing of minimal residual disease from patient-derived xenografts that had been treated with both BRAF and MEK inhibitors has also revealed several transcriptional states, including a neural crest-like state, associated with drug tolerance (Rambow et al., 2018). Although the relationship between these transcriptional states and RAS pathway activity remains unclear, designing pharmaceutical agents targeting resistant states could greatly improve the clinical efficacy of current targeted therapies in melanoma.

The role of RAS pathway alterations in the tumor response to immunotherapies has not yet been fully evaluated but it is conceivable that the mechanisms of sensitivity or resistance to immune checkpoint blockade are largely independent of the activation status of the RAS/MAPK pathway. Yet, by fine-tuning the level of pathway activity and the proliferation rate of melanoma cells, RAS pathway regulators might control the differentiation state of these cells and impact their ability to escape or be recognized by the immune system. The mechanisms of drug resistance appear numerous and complex. Although our understanding of their molecular details has greatly improved over the past 10 years, this knowledge remains to be translated into more-efficient therapies.

### Recent advances and current approaches to drugging the RAS pathway

Because of its smooth external structure and tight GDP/GTP-binding pocket, RAS has long been deemed impossible to target pharmacologically. To circumvent this difficulty, research has focused on direct RAS regulators to better understand the molecular mechanisms of RAS regulation and leverage this knowledge to develop indirect approaches to block RAS activity.

**Fig. 5.**
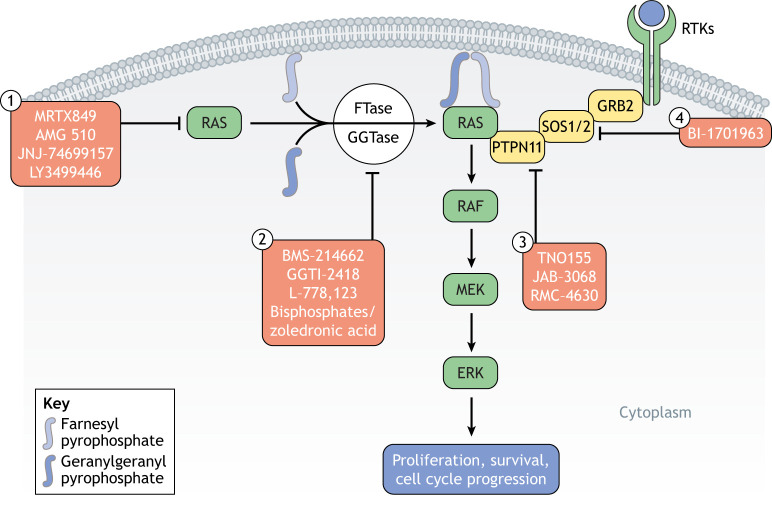
**Direct and indirect RAS inhibitors.** RAS proteins are synthesized in the cytosol, and different covalent inhibitors of KRAS^G12C^ are currently in clinical trials (1). RAS functioning requires membrane anchorage, which can be achieved through farnesylation by farnesyltransferase (FTase) or geranylation by geranylgeranylprenyltransferase (GGTase). By inhibiting FTase or GGTase, RAS membrane anchoring is prevented and RAS function blocked (2). Inhibition of PTPN11 (3) or SOS1/2 (4) prevents RAS activation through upstream signaling.

Farnesyl transferase inhibitors were developed to prevent RAS isoprenylation, a post-translational modification essential for its membrane localization and activity (Hancock et al., 1989; Jackson et al., 1990) (Fig. 5, Table 4). These inhibitors demonstrated strong ability to block aberrant RAS activity *in vitro* and showed promising efficacy in transgenic mouse models of *HRAS*-driven tumors (Kohl et al., 1993, 1995). Farnesyl transferase inhibitors elicited some anti-tumor responses in patients with multiple myeloma or chronic myeloid leukemia, although for the most part, these responses were incomplete and transient (Alsina et al., 2004; Cortes et al., 2003). One limitation to the efficacy of these inhibitors might come from geranylation, an alternative post-translational modification, of KRAS and NRAS (Rowell et al., 1997; Whyte et al., 1997). Inhibitors of geranylgeranyl prenyltransferase-I have subsequently been developed and are currently being tested in combination with farnesyl transferase inhibitors, although these drug combinations might induce significant toxicities.

**Table 4.**
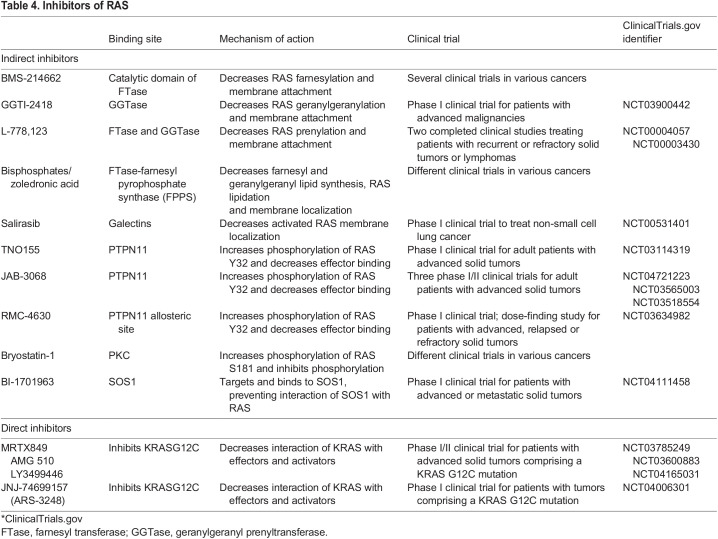
Inhibitors of RAS

Another indirect way to inhibit the functionality of RAS is by preventing its binding to activators or effectors. Several inhibitors have been designed to target the RAS activator PTPN11 (Vainonen et al., 2021) or the interaction between SOS1/2 and RAS (Hillig et al., 2019; Hofmann et al., 2021) (Fig. 5, Table 4). Preclinical studies and clinical trials are ongoing to test the efficacy of these approaches. The nuclear protein serine/threonine kinase 19 (STK19) was recently identified as a new NRAS kinase (Yin et al., 2019). *STK19* is frequently mutated in melanoma and the most-frequent mutation, D89N appears to act as a gain-of-function that amplifies NRAS downstream signaling, promoting melanocyte transformation *in vitro* and *in vivo*. Despite some controversy regarding the mutation annotation of the *STK19* gene (Rodríguez-Martínez et al., 2020), pharmacologic inhibition of STK19 has been suggested to abolish *NRAS*-driven oncogenesis (Yin et al., 2019), a result remarkable enough to warrant further preclinical exploration.

Finally, allosteric inhibitors of RAS, such as MRTX849, AMG510, JNJ-74699157 and LY3499446, have recently made significant progress (Fig. 5, Table 4). Taking advantage of the presence of a new cysteine (C) residue in the G12C KRAS mutant, molecules that can covalently bind C residues were screened using a structure-based approach to identify compounds that could affect RAS conformation (Ostrem et al., 2013). The top hits disrupted switch I and II structures, thus decreasing the affinity of KRAS^G12C^ for GTP and preventing interactions with effectors, such as RAF. Additional compounds similarly favoring the inactive GDP-bound state of the mutant were developed and shown to have strong anti-tumor activity in *KRAS^G12C^* tumor cell lines (Lito et al., 2016; Patricelli et al., 2016). Some of these allosteric inhibitors were then successfully tested in mouse xenograft models and in patients (Canon et al., 2019; Janes et al., 2018). Strikingly, phase I and II clinical trials of these inhibitors recently reported >30% response rates among patients with *KRAS^G12C^*-driven non-small cell lung cancer, with a median duration of response of 11 months (Hong et al., 2020; Skoulidis et al., 2021). Although not the most common *KRAS* mutation, G12C is found in 13% of patients with non-small cell lung cancer. These results open new perspectives for the design of small molecules targeting RAS in a conformation-specific manner, although the mutant residue in other RAS mutants – including NRAS^Q61R^ that is most frequently found in melanoma – might not offer the same opportunities for covalent binding as the C residue in KRAS^G12C^.

## Future avenues and concluding remarks

Forty years after the discovery of the *RAS* proto-oncogene, much progress has been made in our understanding of its roles in normal cell biology and cancer. Key players of the RAS pathway have been identified and regulatory mechanisms have been elucidated. These advances have allowed the design of drugs that target RAS regulators or effectors, some of which have shown antitumor efficacy in the clinic. However, the example of melanoma suggests that these approaches are likely to be met with drug resistance. This has prompted a wave of research into resistance mechanisms and combinations of drugs targeting the RAS pathway at different levels. These have shown promise and could become the strategy of choice in the near future, provided associated toxicities remain manageable. Yet, there is a need to further understand protein–protein interactions between RAS and its regulators to design new compounds capable of interfering with RAS functions, with the goal to develop orthogonal therapies against RAS-driven tumors. Recent years have demonstrated that there is still room to discover important players and new feedback loops within the pathway, as well as additional roles for RAS pathway regulators in cancer. Naturally, the hunt for molecules that can directly target RAS is ongoing and the example of KRAS^G12C^ inhibitors has raised new hopes that RAS is not ‘undruggable’ for much longer. Although several RAS mutants have been documented in cancer, structure-based design of allosteric inhibitors impeding the main mutants would represent a tremendous step towards effective clinical management of patients with RAS-driven cancer. Additionally, evidence from melanomas indicates that the genetic plasticity of human tumors as well as their heterogeneity are a source of drug tolerance and relapse. Different genetic subtypes are likely to require different treatments and testing more drug combinations in preclinical models of RAS pathway-dependent tumors could uncover new therapeutic synergies. Developing models that more accurately and more completely recapitulate the genetic complexity of RAS-driven tumors might, thus, constitute one of the greatest challenges in today's cancer research.
